# Acute infarction after mechanical thrombectomy is better delineable in virtual non-contrast compared to conventional images using a dual-layer spectral CT

**DOI:** 10.1038/s41598-018-27437-7

**Published:** 2018-06-19

**Authors:** Isabelle Riederer, Alexander A. Fingerle, Thomas Baum, Jan S. Kirschke, Ernst J. Rummeny, Peter B. Noël, Daniela Pfeiffer

**Affiliations:** 1 Department of Diagnostic and Interventional Radiology, Klinikum rechts der Isar, Technische Universität München, Munich, Germany; 2 Department of Diagnostic and Interventional Neuroradiology, Klinikum rechts der Isar, Technische Universität München, Munich, Germany

## Abstract

The aim was to evaluate Virtual Non-Contrast (VNC)-CT images for the detection of acute infarcts in the brain after mechanical thrombectomy using a dual-layer spectral CT. 29 patients between September 2016 and February 2017 with unenhanced head spectral-CT after mechanical thrombectomy and available follow-up images (MRI, n:26; CT, n:3) were included. VNC-CT and conventional CT (CT) images were reconstructed using dedicated software. Based on those, contrast-to-noise ratio (CNR), and the volume of infarction were measured semi-automatically in VNC-CT, CT and MRI. Furthermore, two readers independently assessed the VNC-CT and CT images in a randomized order by using the ASPECT score, and inter-rater reliability, sensitivity and specificity were calculated. CNR was significantly higher in VNC-CT compared to CT (3.1 ± 1.5 versus 1.1 ± 1.1, p < 0.001). The mean estimated volume of infarction was significantly higher in VNC-CT compared to CT (72% versus 55% of the volume measured in MRI, p < 0.005). Inter-rater reliability was higher in VNC-CT compared to CT (0.751 versus 0.625) and sensitivity was higher in VNC-CT compared to CT (73% versus 55%). In conclusion, acute ischemic lesions after mechanical thrombectomy are better definable in VNC-CT compared to CT images using a dual-layer spectral CT system.

## Introduction

In the last years, spectral imaging methods^1^ have been increasingly used in research and clinical practice to evaluate simultaneously anatomy and tissue composition. There are different dual-energy spectral imaging approaches: e.g. two different scans using different kVp (e.g. 80 and 140 kVp), one fast scan switching between two different kVp^2^, or dual x-ray sources^3^. Recently, a novel type of spectral dual-energy imaging approach has been introduced, which is based on a dual-layer detector system that identifies photons of low and high energy simultaneously^4^. This system is able to acquire spectral data in each CT scan per default without additional radiation doses or the need to alter x-ray tube parameters. All spectral CT data can be analyzed retrospectively without special scan protocols. Post-processing using a dedicated software can generate Virtual Non-Contrast (VNC)-CT images without iodine, and iodine density overlay maps.

Unenhanced head CT is commonly performed after mechanical thrombectomy of ischemic stroke patients to assess for early complications such as haemorrhage^5^. However, the differentiation between haemorrhage and extravasation of iodinated contrast agent, due to a disrupted blood brain barrier, is often difficult, as they exhibit similar Hounsfield units (HU). Some studies have already shown that this differentiation is possible with dual-energy spectral CT^6–8^. Another point of interest, while evaluating control CT scans after mechanical thrombectomy, is the early assessment of infarction development for prediction of outcome and adjustment of therapy management. The larger the ischemic area is, the higher the risk of future haemorrhage^9^. Often it is difficult to delineate acute infarction in unenhanced CT, especially in the early stages due to low CNR, and follow-up images or diffusion-weighted images in magnetic resonance imaging (MRI) are necessary, which could delay diagnosis. Other investigates illustrated in initial studies that delineation of acute infarction can be performed by analyzing VNC-CT compared to CT images using dual-energy CT^10,11^.

The purpose of our study is to evaluate the potential of dual-layer detector spectral CT for the detection of acute infarction in the brain after mechanical thrombectomy by comparing VNC-CT and CT images.

## Results

### Contrast-to-noise-ratio

**Figure 1:**
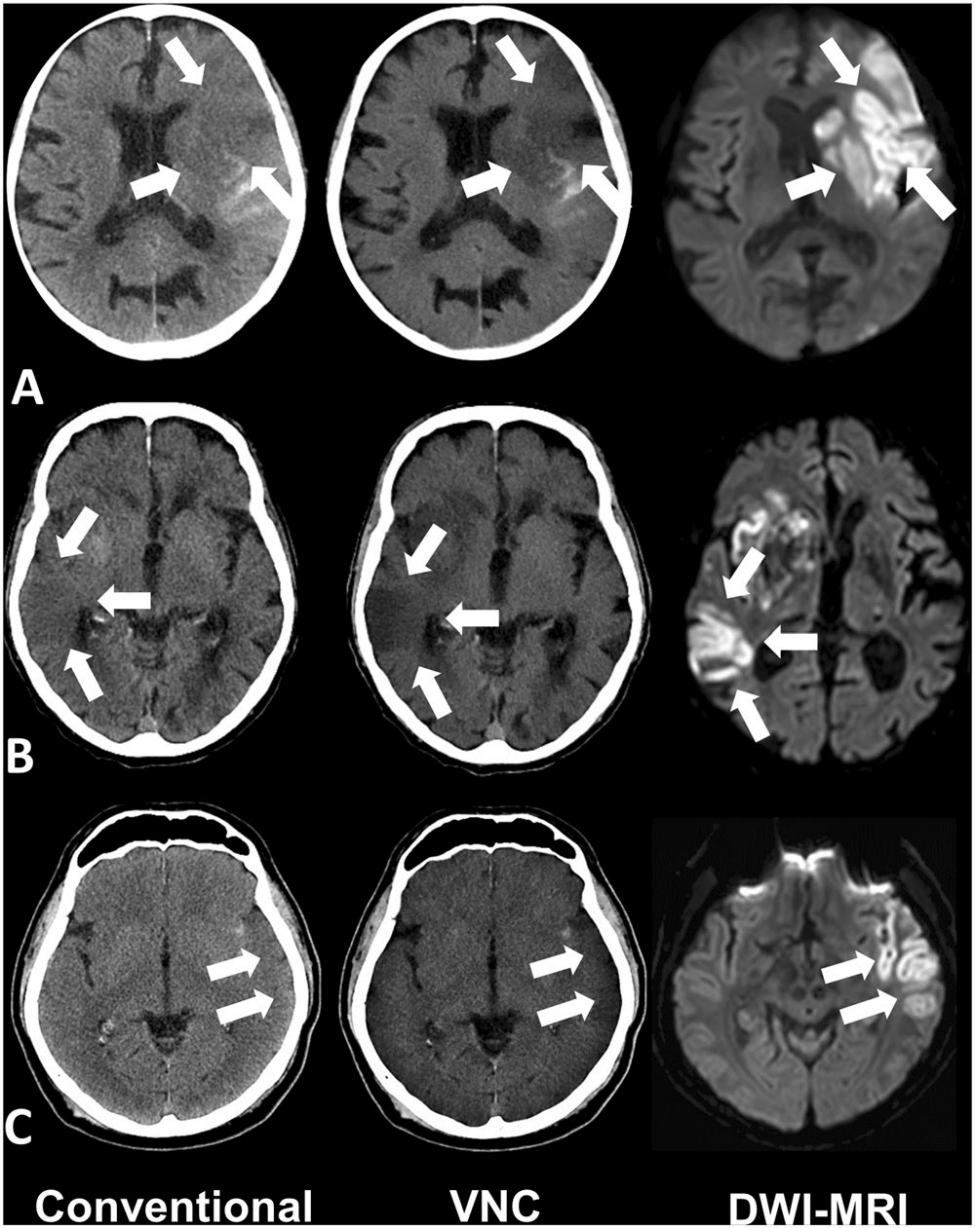
Examples of unenhanced head spectral CT images of patients after mechanical thrombectomy. Please note the acute developing infarctions (white arrows), which are better visible in Virtual Non-Contrast (VNC)-CT images (in the middle) compared to conventional images (left). Follow-up MRI as gold standard on the right side with (A) 2.9 d; (B) 1.5 d and (C) 2.4 d after spectral CT. (A) 49 year old male 13 h after successfull revascularization (TICI 3) of a thrombus in the left M1-Segment. (B) 88 year old male patient 12 h after successfull revascularization (TICI 3) of a thrombosis in the terminal carotid segment and M1-Segment on the right side. (C) 80 y old male patient 12 h after successfull revascularization (TICI 2b) of a thrombosis in the M1-Segment on the left side.

Acute ischemic lesions appear more hypodense in VNC-CT compared to CT images with significantly higher CNR (VNC-CT: 3.1 ± 1.5 compared to CT: 1.1 ± 1.1, p < 0.001). Figure 1 illustrates examples of three patients after mechanical thrombectomy. Please note the developing infarctions (white arrows), which are clearer visible in VNC-CT images compared to CT images.

### Volume measurements

The mean estimated volume of infarction was significantly higher in VNC-CT compared to CT images (VNC-CT: 28 ± 51 cm³ versus CT: 21 ± 49 cm³; 72% versus 55% of the volume measured in MRI, p < 0.005).

### ASPECT Score

The inter-rater reliability between reader 1 and 2 using cohen’s kappa was higher in VNC-CT compared to CT images (VNC-CT: 0.751 versus CT: 0.625 (ASPECT); VNC-CT: 0.726 versus CT: 0.686 (pc-ASPECT). The inter-rater reliability for each reader comparing VNC-CT or CT images with the gold standard DWI-MRI for the ASPECT or pc-ASPECT score was also higher in VNC-CT compared to CT images (Table 1), with fair agreement between CT and DWI-MRI (κ = 0.23) and moderate agreement between VNC-CT and DWI-MRI (κ = 0.51) for ASPECT score and almost comparable results for pc-ASPECT (κ = 0.39–0.45).

**Table 1:** Inter-rater reliability (cohens kappa) for comparison between Virtual Non-Contrast (VNC)-CT and conventional CT images with goldstandard DWI-MRI for reader 1 and 2 separately.

		ASPECT	pc-ASPECT
VNC	Reader 1	0.511	0.447
	Reader 2	0.514	0.442
CT	Reader 1	0.232	0.440
	Reader 2	0.234	0.391

### Sensitivity and specificity

Table 2 lists the sensitivity and specificity values for the detection of acute infarction by both readers analyzing VNC-CT or CT images, separately for the ASPECT score and pc-ASPECT score. The sensitivity was higher in VNC-CT compared to CT images for both readers [VNC-CT: 73% [95% CI: 64–80] versus CT: 54–55% [95% CI: 45–64]. The specificity was 99–100% [95% CI: 98–100] in both VNC-CT and CT images for both readers.

**Table 2:** Sensitivity and specificity of Virtual Non-Contrast (VNC)-CT and conventional CT images for reader 1 and 2.

ASPECT	Sensitivity [95% CI]	CT	Specificity [95% CI]	CT
	VNC-CT		VNC-CT	
Reader 1	79% [70–86]	60% [50–70]	99% [96–100]	99% [97–100]
Reader 2	78% [69–86]	56% [46–66]	99% [96–100]	98% [95–100]
pc-ASPECT				
Reader 1	40% [19–64]	26% [9–51]	100% [98–100]	100% [99–100]
Reader 2	40% [16–68]	38% [15–64]	100% [99–100]	100% [99–100]
Total				
Reader 1	73% [64–80]	55% [46–64]	99% [98–100]	100% [99–100]
Reader 2	73% [64–80]	54% [45–64]	100% [98–100]	99% [98–100]

## Discussion

The results of our study show that VNC-CT images produced by a dual-layer spectral CT enable a better delineation of acute infarction after mechanical thrombectomy compared to conventional CT images with significantly higher CNR, volume estimation compared to the gold standard DWI-MRI, and higher sensitivity and better inter-rater reliability.

The results of our study are generally in conformance with two previous studies analyzing VNC-CT images of dual-energy CTs^10,11^. They concluded that VNC-CT images allow better detection of acute ischemia than conventional CTs. The novelty of our study is the first use of a dual-layer spectral CT that has the advantage to acquire spectral data without additional radiation doses or alteration of x-ray tube parameters. The radiation dose of the head CT scan in our study was relatively low with 1.9 mSv, compared to the other studies where the radiation dose was 2.3 mSv^10^. A major difference which could have an influence of stroke detection in CT is the exact time point of the CT scan. In our study, the control CT scans were performed around 12 h after the interventional procedure (13 ± 6 h, according to the standard operation procedure of our hospital), whereas other studies evaluated stroke delineation in DECT 24 h^11^ and 1h^10^ after mechanical thrombectomy. In our study, we have seen a very good agreement for the estimation of ischemic brain volume using DECT and MRI as a gold standard. Additionally, we also included patients with an occlusion of the basilar artery and added the pc-ASPECT Score for the analysis of the posterior circulation.

The better delineation of infarction in VNC-CT images compared to CT images could be explained due to the disrupted blood brain barrier of ischemic lesions with extravasation of iodinated contrast agent, which is injected intra-arterially during the recanalization procedure. The contrast agent could mask the soft hypodensity of ischemic lesions in the conventional CT in the early stages whereas the hyperdense signal of the iodinated contrast agent disappears in VNC-CT images and reveal the developing infarction. As ischemic lesions appear more hypodense in VNC-CT images compared to conventional CT images, the contrast between ischemic lesions and non-affected tissue is higher in VNC-CT images. The noise of the non-affected tissue is comparable in conventional and VNC-VT images. This effect is desirable and improves delineation of infarction. In accordance to this consideration, the calculated CNR was higher in VNC-CT images compared to conventional CT images.

One group^12^ studying the contrast enhancement and contrast extravasation in ischemic stroke patients after intraarterial thrombolysis described a persistence of the extravasated contrast medium in the 24 h control CT. Therefore, it seems assumable that the iodinated contrast agent rests for some time in the brain tissue and is not resorbed within the next 12 h – the timepoint of our CT scans. This seems to be a good explanation for the pronounced hypodensity of ischemic lesions in the VNC-CT images.

With this technique, developing ischemic lesions can be depicted earlier compared to conventional CT and may improve therapy management and prediction of outcome. Additionally, in the future, imaging might become even more sensitive with photon-counting CT. First results of photon-counting CT scans of the brain have recently been published^13^ showing greater gray-white matter contrast compared with conventional CT. Furthermore, the improvement of the delineation of ischemic lesions might have an impact on studies comparing the outcome of different recanalization techniques as the estimated infarction volume is closer to the reality using VNC-CT compared to conventional CT.

Limitations: This study has a retrospective design and the total number of patients was relatively small. Another limitation was the temporal delay of 2.4 days between CT and follow-up MRI as gold standard. During this delay, the ischemic areas could have expanded.

To conclude, acute ischemic lesions after mechanical thrombectomy are better definable in VNC-CT images compared to CT images as produced by a dual-layer spectral CT with significantly higher CNR, sensitivity, volume estimation and higher inter-rater reliability.

## Material and Methods

### Patients

Between September 2016 and February 2017, 56 patients underwent unenhanced computed tomography (CT) of the head within the standard clinical protocol as a matter of routine. Within this group, 29 patients had been examined after mechanical thrombectomy (13 ± 6 h) (15 female, 14 male, age range 42–90 y, mean age 72 ± 14 y), having available follow-up imaging (MRI, n: 26; CT, n: 3; 57 ± 46 h after spectral CT). The latter group of 29 patients were included into our study, data were anonymized and analyzed retrospectively with the approval of the local ethics committee. The study was conducted in accordance with the 2013 revised Declaration of Helsinki. The pattern of vessel occlusion was in 24 cases in the anterior circulation (ICA or terminal carotid segment, n: 7; M1, n: 13; M2, n: 4) and in 5 cases in the posterior circulation (basilar artery) with TICI score “3/2b” in 28 cases, and “0” in 1 case.

### Imaging acquisition

CT was performed on a dual-layer spectral CT (IQon spectral CT, Philips Healthcare, USA) with 120 kVp and 260 mAs. Post-processing was performed using a dedicated software (IntelliSpace Portal v6.5.0.02901, Philips, Healthcare, USA). The VNC-CT images without iodine and the conventional CT images were generated from spectral data sets, reformatted with 5 mm slice thickness and reoriented in a standard manner in the anterior commissure – posterior commissure plane. For both, the conventional CT and VNC-CT images, we used a sharp filter kernel for the brain (UC). The conventional CT images were reconstructed using iterative reconstruction (iDose4), whereas the VNC-CT images were reconstructed using a special spectral reconstruction mode. In both cases, level 2 was used, therefore the images were considered comparable and image analysis was performed. Follow up images were available of a 3 T MRI (Philips, Achieva) or a conventional CT (Brilliance, Philips Healthcare, USA; Somatom, Siemens Healthineers, Germany). The effective radiation dose was calculated by multiplying the dose-length product (840 [mGy*cm]) by a conversion coefficient (0.0023 mSv mG-1cm-1) for the head according to European guidelines (Quality criteria for computed tomography, EU report 16262. Luxembourg: Commission of the European Communities, 1999, available from http://www.drs.dk/guidelines/ct/quality). The effective radiation dose was 1.9 mSv.

### Imaging analysis

The imaging data were analyzed retrospectively on a standard PACS workstation (Sectra Workstation IDS7, Version 17.1.18.3596, Sectra Healthcare). Small circular region of interest (ROI) (8–12 mm²) were drawn in the centre of the ischemic area in CT and VNC-CT images consulting additionally diffusion-weighted images of follow-up MRI or follow-up CT and in the corresponding contralateral unaffected brain tissue in order to measure mean densities of the Hounsfield units (HU). We carefully tried to avoid areas of haemorrhagic transformations or iodine extravasation. Furthermore, contrast-to-noise ratio (CNR) was calculated according to following formula:

$${\rm{CNR}}=({{\rm{S}}}_{{\rm{lesion}}}-{{\rm{S}}}_{{\rm{non}} \mbox{-} {\rm{affected}}{\rm{tissue}}})/{{\rm{SD}}}_{{\rm{non}} \mbox{-} {\rm{affected}}{\rm{tissue}}},$$

where S_lesion_ and S_non-affected tissue_ represent the mean signal in a ROI in the ischemic lesion and normal-appearing brain tissue in the corresponding contralateral side, respectively. SD_non-affected tissue_ is the standard deviation of the normal-appearing brain tissue in the contralateral side.

Furthermore, the volume of infarction was estimated by one reader in VNC-CT and CT images and MRI or follow-up CT in a randomized order, semi-automatically using the IS Portal (IntelliSpace Portal v. 5.02.40009, 11. Sep 14, Philips Healthcare Netherlands).

Additionally, two experienced readers independently assessed the VNC-CT and CT images in a randomized order regarding the presence of acute ischemic lesions using the ASPECT score^14^ for infarction in the area of the medial cerebral artery or the posterior circulation (pc)-ASPECT score^15^ for the territory of the basilar artery. Sensitivity and specificity were calculated.

### Statistical analysis

Paired student’s t-test was used for the comparison of the CNR and differences between estimated volume measurements. Significant differences were defined by a p < 0.05. Cohen’s kappa was calculated to determine inter-observer variability of the qualitative evaluation using the (pc-) ASPECT scores, with values < 0 indicating no agreement; 0–0.20: slight; 0.21–0.40: fair; 0.41–0.60: moderate; 0.61–0.80: substantial and 0.81–1.0: almost perfect agreement.

### Data Availability

The datasets analyzed during the current study are available from the corresponding author on reasonable request.
